# Pathogenic *EDA* Mutations in Chinese Han Families With Hypohidrotic Ectodermal Dysplasia and Genotype–Phenotype: A Correlation Analysis

**DOI:** 10.3389/fgene.2020.00021

**Published:** 2020-02-04

**Authors:** Yang Han, Xiuli Wang, Liyun Zheng, Tingting Zhu, Yuwei Li, Jiaqi Hong, Congcong Xu, Peiguang Wang, Min Gao

**Affiliations:** ^1^Department of Dermatology of First Affiliated Hospital, First Affiliated Hospital of Anhui Medical University, Hefei, China; ^2^Institute of Dermatology, Anhui Medical University, Hefei, China

**Keywords:** hypohidrotic ectodermal dysplasia, whole-exome sequencing, Sanger sequencing, ectodysplasin A gene, gene mutation

## Abstract

**Background:**

This study aimed to investigate the genetic causes of hypohidrotic ectodermal dysplasia (HED) in two families and elucidate the molecular pathogenesis of HED in Chinese Han patients.

**Methods:**

Whole-exome sequencing (WES) was used to screen HED-related genes in two family members, followed by confirmatory Sanger sequencing. Bioinformatics analysis was performed for the mutations. We reviewed HED-related articles in PubMed. **χ**^2^- and Fisher's tests were used to analyze the genotype–phenotype correlations.

**Results:**

(1) WES identified *EDA* missense mutations [c.1127 C > T (p.T376M; NM_001005609)] in family 1 and an *EDA* nonframeshift deletion mutation [c.648_683delACCTGGTCCTCCAGGTCCTCCTGGTCCTCAAGGACC (p.216_228delPPGPPGPPGPQGP; NM_001005609)] in family 2. Sanger sequencing validated the results. ANNOVAR (ANNOtate VARiation) annotation indicated that c.1127 c > T was a deleterious mutation. (2) The review of published papers revealed 68 novel mutations related to HED: 57 (83.8%) were *EDA* mutations, 8 (11.8%) were *EDAR* mutations, 2 (2.9%) were *EDARADD* mutations, 1 (1.5%) was a *WNT10A* mutation, 31 (45.6%) were missense mutations, 23 (33.8%) were deletion mutations, and 1 (1.5%) was an indel. Genotype–phenotype correlation analysis revealed that patients with *EDA* missense mutations had a higher frequency of hypohidrosis (P = 0.021).

**Conclusions:**

This study identified two *EDA* gene mutations in two Chinese Han HED families and provides a foundation for genetic diagnosis and counseling.

## Introduction

Ectodermal dysplasias (EDs) are genetically heterogeneous diseases caused by developmental failure in two or more ectodermal structures such as teeth, sweat glands, hair, nails, and skin. The most frequent subtype is hypohidrotic ectodermal dysplasia (HED) with a prevalence of ~1/100,000 (Wisniewski et al., 2002). HED includes autosomal dominant (AD), autosomal recessive (AR), and X-linked forms. Among these, X-linked HED (XL-HED, MIM #305100) is the most common form and is caused by mutations in the *EDA g*(Ectodysplasin A, MIM 300451) gene (Kere et al., 1996).

HED (also known as Christ-Siemens-Touraine syndrome) is characterized by hypohidrosis (reduced ability to sweat), hypotrichosis (sparseness of scalp and body hair), and hypodontia (congenital absence of teeth) (https://www.ncbi.nlm.nih.gov/books/NBK1112/). In addition to the above clinical characteristics, HED can also be complicated with atopic diathesis (hypohidrosis or anhidrosis itself might impair the skin barrier) (Koguchi-Yoshioka et al., 2015), eczema, upper airway infections (Monroy-Jaramillo et al., 2017), impaired breast development (more common in females) (Wahlbuhl-Becker et al., 2017), and other conditions. Homozygous male patients usually have typical clinical manifestations of hypodontia, hypohidrosis, and sparse hair and characteristic facial features including frontal bossing, chin prominence, saddle nose, wrinkles, low-set ears, maxillary hypoplasia, and periorbital hyperpigmentation (Namiki et al., 2016; Liu et al., 2018a). Heterozygous female carriers usually have a mild clinical phenotype with sparse hair or teeth and abnormal tooth morphology (peg-shaped teeth), but severe clinical characteristics have also been observed in females (associated with extremely skewed X-chromosome inactivation) (Lei et al., 2018).

In this study, we report two *EDA* gene mutations—a pathogenic missense mutation and a deletion mutation—in two Chinese Han HED families. Gene functional annotation was used to predict the pathogenicities of the detected mutations.

## Materials and Methods

### Clinical Sample

The study was approved by the Ethical Review Committee of Anhui Medical University and was performed in adherence with the principles of the Declaration of Helsinki. All participants or their guardians signed written informed consent forms. Based on the genetic pattern and the proband's clinical manifestations, the preliminary diagnosis of HED was made by the chief dermatologist.

#### Family 1

**Figure 1:**
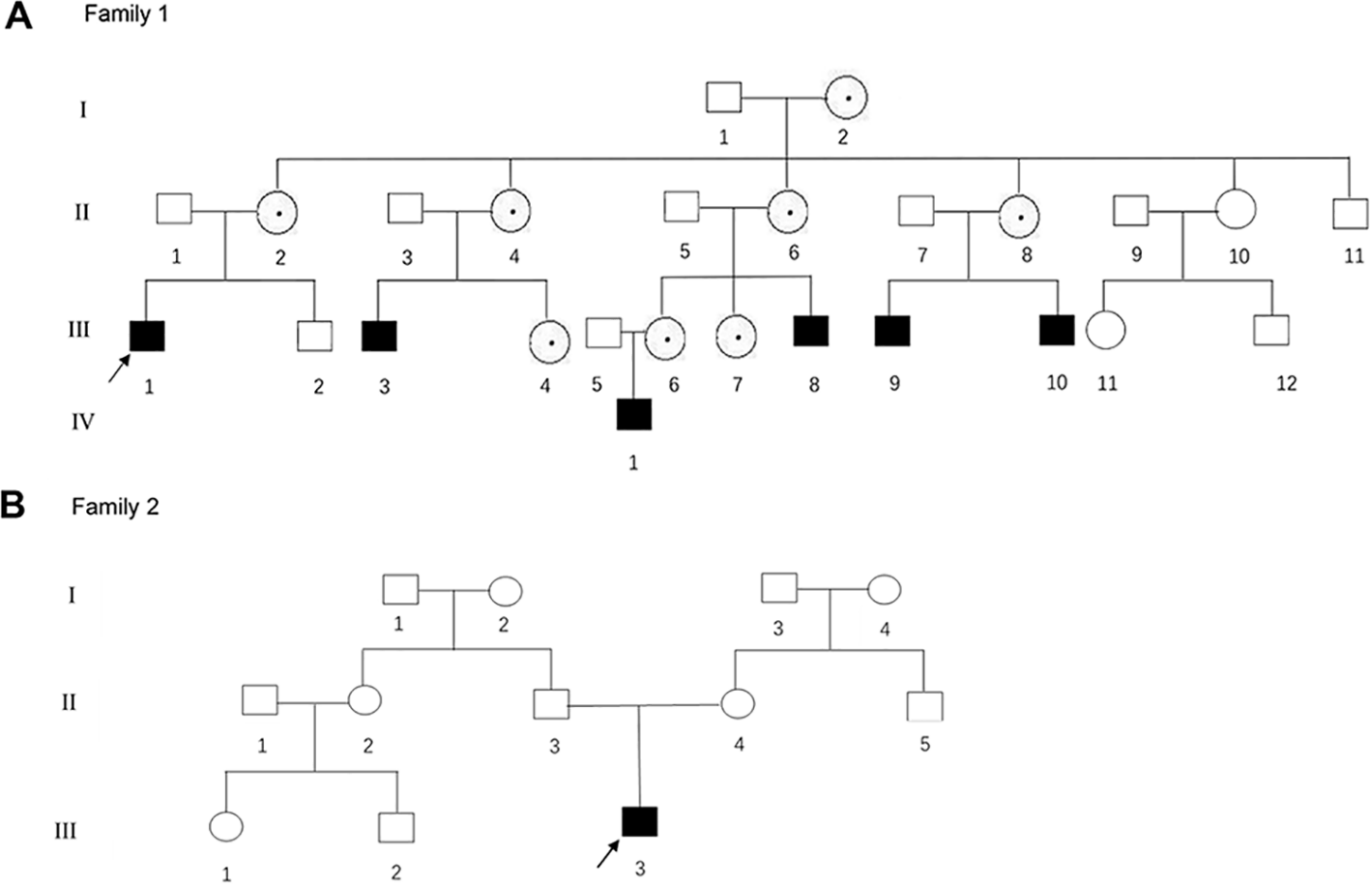
**(A**, **B)** The pedigree gram of two Chinese HED cases. The proband was marked with the arrow. Males were indicated by squares; Females were indicated by circles. Blackened symbols represented male patients who were carried the mutations through mutation sequencing. The circles with black dots represent the female carriers.

**Figure 2:**
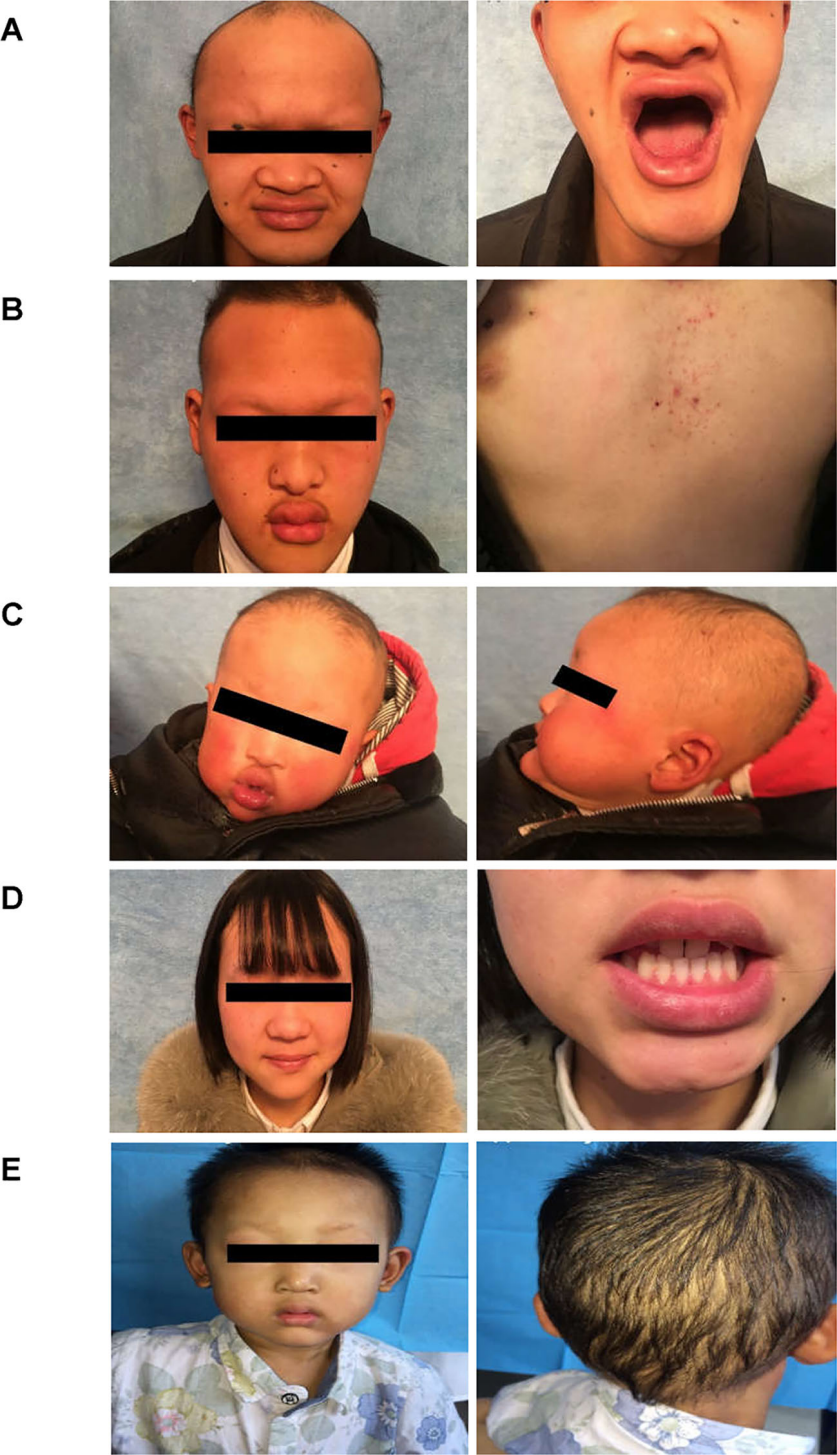
Clinical representations of two Chinese HED family members. **(A)** Sparse hair, saddle nose, protuberant lips, and hypodontia (Family 1 III:1). **(B)** Sparse hair, hypohidrosis, hypodontia, protuberant lips and dry skin. The patient presented with mild eczematoid dermatitis on the chest (Family 1 III: 8). **(C)** Typical HED appearance, born with sparse hair, no sweat, hypodontia (Family 1 IV:1). **(D)** Female carriers (Family 1 III:7) showed no abnormalities except for sparse teeth and abnormal morphology (peg-shaped teeth). **(E)** Proband of family 2, 4-year-old boy, with sparse hair, missing teeth, frontal bossing, prominent lips, presenile manifestations and periorbital wrinkling.

The proband of family 1 was a 28-year-old male (**Figures 1A** **and 2A**) who was born with hypotrichosis, hypodontia, hypohidrosis, dry skin, normal intelligence, and dry nasal mucosa. His brother was normal, and his mother had no abnormal pregnancy history. The wife of the proband had a history of miscarriage. The male patients with the mutation in this family all have very typical HED facial features, with clinical characteristics of hypotrichosis, hypodontia, hypohidrosis, and partial peg-shaped teeth (III;:3; III;:9). One person has eczema (III:8, **Figure 2B**), and only IV:1 (**Figure 2C**) shows mental retardation. The main clinical characteristics of female mutation carriers in this family are sparse teeth and abnormal morphology; only III:7 (**Figure 2D**) has a saddle nose and peg-shaped teeth. The clinical features of families 1 and 2 are summarized in **Table 1**.

**Table 1:** Clinical features of members in each family.

Family	Person ID	Gender	Age	Facial features	Thin or wrinkled skin	Sparse or curly hair	Hypohidrosis	Tooth loss	Eczema	Others
1	I:2	F	69	**−**	**−**	**−**	**−**	**−**	**−**	Teeth sparse
	II:2	F	49	**−**	**−**	**−**	**−**	**−**	**−**	Teeth sparse
	II:4	F	47	**−**	**−**	**−**	**−**	**−**	**−**	Teeth sparse
	II:6	F	44	**−**	**−**	**−**	**−**	**−**	**−**	Teeth sparse
	II:8	F	37	**−**	**−**	**−**	**−**	**−**	**−**	Teeth sparse
	III:1	M	28	**+**	**+**	**+**	**+**	**+**	**−**	Dysspermia
	III:3	M	20	**+**	**+**	**+**	**+**	**+**	**−**	Peg-shaped teeth
	III:4	F	12	**−**	**−**	**−**	**−**	**−**	**−**	Teeth sparse; myopia
	III:6	F	24	**−**	**−**	**−**	**−**	**−**	**−**	Teeth sparse
	III:7	F	18	Saddle nose	**−**	**−**	**−**	**−**	**−**	Peg-shaped teeth
	III:8	M	16	**+**	**+**	**+**	**+**	**+**	**+**	—
	III:9	M	16	**+**	**+**	**+**	**+**	**+**	**−**	Peg-shaped teeth and hypopigmentation
	III:10	M	8	**+**	**+**	**+**	**+**	**+**	**−**	—
	IV:1	M	2	**+**	**+**	**+**	**+**	**+**	**−**	Abnormal intelligence
2	III:3	M	4	**+**	**+**	**+**	**+**	**+**	**−**	Skin hyperpigmentation and hypopigmentation

F, female; M, male; +; feature present; **–**, within normal clinical limits.

#### Family 2

A 4-year-old Chinese boy presented with HED (**Figure 1B**) (proband, **Figure 2E**). Clinical characteristics included dry skin, decreased sweating, sparse hair, missing teeth, frontal bossing, prominent lips, periorbital wrinkling, and presenile manifestations. He was intolerant to heat. Patchy pigmentation and depigmentation were observed on his trunk and limbs. His parents were normal.

### Mutation Detection and Bioinformatics Analysis

#### Peripheral Blood Collection and DNA Extraction

Peripheral blood (3–5 mL) was collected from members of both families. Genomic DNA was extracted from the peripheral blood lymphocytes by standard procedures using Qiagen genomic DNA extraction kits (No: 51206; Qiagen, Hilden, Germany) and stored at −80°C until testing.

#### Whole-Exome Sequencing (WES)

Qualified genomic DNA samples (four affected and five unaffected individuals from two families: Family 1-III:1.3.9 (patients); II:11, III:2.12 (normal relatives) and Family 2-III:3 (patient); II:3.4 (normal relative) were analyzed by WES. After qualified quality control, we used the BGISEQ-500 for sequencing of each qualified library. In the comparison on the target area, an approximately 60.33-Mb-long target area was captured, and clean reads of each sample were aligned to the human reference genome sequence (GRCh38/HG38) using Burrows-Wheeler Aligner (BWA V0.7.15). The average sequencing depth of the target region was approximately 156.77X. Single nucleotide polymorphisms (SNPs) and insertions and deletions (indels) were identified by the Genome Analysis Toolkit (GATK v3.7). For mutation detection, information on previously reported pathogenic genes was first analyzed. If no pathogenic mutations were found in the previously reported genes, the possible pathogenic mutations were searched in the previously reported linkage region. If no pathogenic mutations were found in any of the above cases, the search area was enlarged to the entire exome, and the disease-related harmful mutations or genes were screened out through analysis strategies based on sample situation, the harmfulness of variation, and gene function and phenotype. The following criteria can be referred to: 1) This mutation is not in the genome repeat region (genomicSuperDups and repeat have no annotation information); 2) The frequency in the 1000 Genome Project is <0.01; 3) This mutation is located in the exonic or splicing region and missense, splicing, indel, and other variation types that may affect the protein are selected; 4) selection of variation type according to heredity pattern: heterozygous variation type for AD inheritance, homozygous variation or compound heterozygous mutation type for AR inheritance, and co-separation of genotypes and phenotypes consistent with case-control genotypes in the family (common in both cases and none in control); 5) the mutation was predicted as pathogenic by SIFT, Polyphen, MutationTaster, and CADD. After the above analysis, a small number of pathogenic mutations were identified, which required sequencing in the family, between families, distributed samples, and normal populations.

#### Sanger Sequencing

We used Sanger sequencing to validate the mutations. The primers of all coding exon and intron-exon boundaries of the *EDA* gene were designed by Mapbioo Biotech Co. Ltd. (Shanghai, China). After amplification, the polymerase chain reaction products were purified with a Universal DNA Purification Kit (DP214-03; Tiangen, Beijing, China) and sequenced on an ABI 3730xl automated sequencer. The sequencing results were analyzed using DNA sequencing analysis software, interpreted using Sequencing Analysis 5.2.0, and compared and analyzed using Sequencher 5.1.

#### Mutation Functional Annotation by ANNOVAR

By ANNOVAR annotation, the specific position of the mutations and the values of SIFT and Polyphen2_HVAR can be obtained to annotate the pathogenicity. A SIFT score <0.05 predicts pathogenicity. Polyphen2_HVAR contains two values. The first is the PolyPhen2 score, and a higher value indicates it is more “harmful,” that is, the SNP is likely to cause changes in protein structure or function. The second is D, P, or B [D: probably damaging (≥0.909), P: potentially damaging (0.447≤pp2_hvar ≤ 0.909), B: benign (pp2_hvar ≤ 0.446)].

### Literature Review and Statistical Analysis

Papers reporting *EDA* mutations in PubMed (http://www.ncbi.nlm.nih.gov/pubmed/) published between January 1, 2015, and February 3, 2019, were collected. One article reviewed (Guazzarotti et al., 2015) did not give specific mutations, so we did not summarize it, but those with records about different clinical characteristics (typical HED facial features, hypotrichosis, hypohidrosis, hypodontia/oligodontia) in patients with *EDA* missense mutations, *EDA* deletion mutations, and *EDAR* mutations were included. All statistical analyses were performed with SPSS version 16.0 software (SPSS, Chicago, IL, USA). Statistical significance was determined by χ^2^ and Fisher's tests. The level of statistical significance was set at 5% (P < 0.05).

## Results

### WES

WES was performed on nine DNA samples, and each sample was sequenced on average of 18,683.88 Mb of raw bases. After removing low-quality reads, an average of 186,782,461 reads was obtained per sample of clean reads (18,676.67 Mb). The average GC content was 50.33%. Clean reads from each sample were aligned to the human reference genome sequence (GRCh38/HG38), and an average of 99.82% of reads were aligned to the reference genome. Duplicate reads were removed, and an average of 159,952,948 effective reads was obtained. Overall, 59.51% of the effective bases were within the target area. The average sequencing depth of the target region was approximately 156.77X, with an average of 99.71% of the target region covered by at least one read and 99.21% of the target region covered by at least 10 reads. Overall, the average number of newly discovered SNPs in all samples was ~1,000, and the disease-causing gene identified through screening was *EDA*.

### Sanger Sequencing of the *EDA* Gene

**Figure 3:**
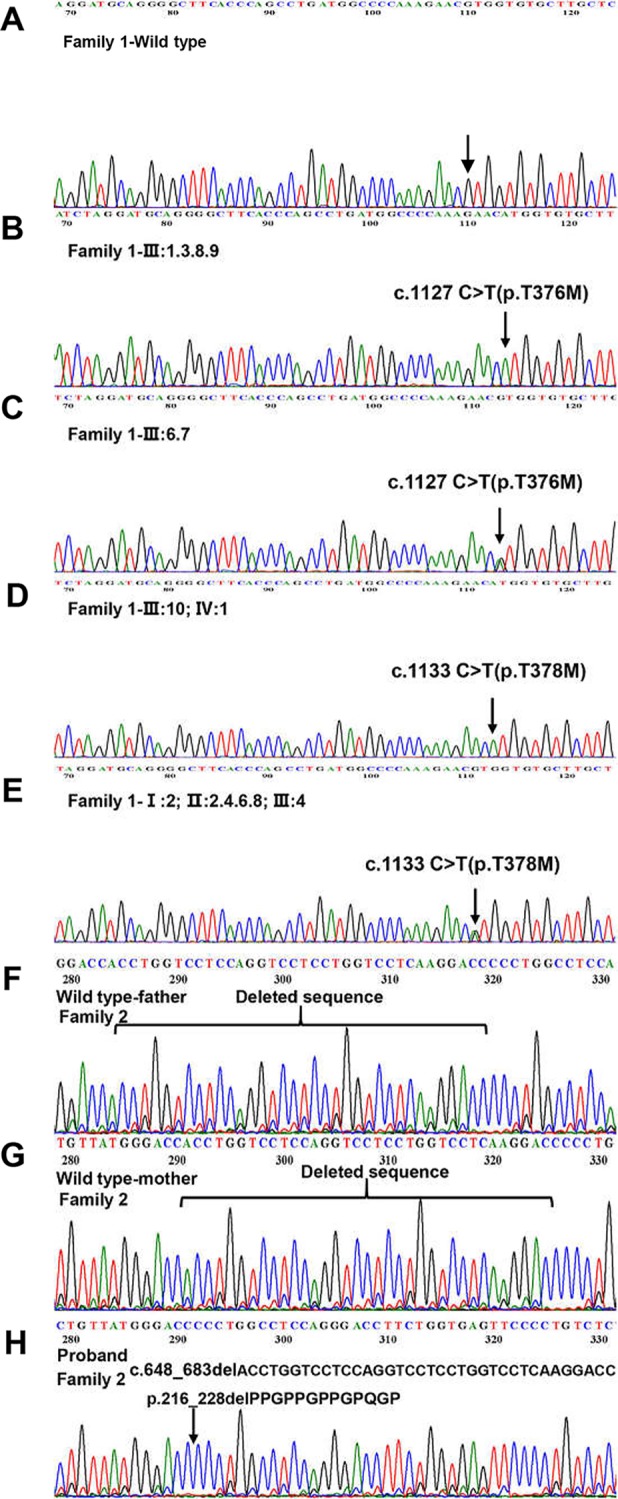
Detection of *EDA* mutations in two family. **(A)** Family 1-Wild type. **(B**, **D)** Homozygous variant identified in Family 1. **(C**, **E)** Heterozygous variant identified in Family 1. **(F)** Family 2-Wild type (proband’s father). **(G)** Family 2-Wild type (proband’s mother). **(H)** The 36 kb deletion mutation from the proband (family 2 III:3). The location of the bases missing from the proband has been marked with a black arrow.

We sequenced 15 affected and nine unaffected members from two families and identified two missense mutations (these were identical mutations located in different transcripts) in family 1 (**Table 2**): c.1127 C > T (p.T376M; NM_001005609; *EDA* transcript variant 2) and c.1133 C > T (p.T378M; NM_001399.5; *EDA* transcript variant 1). c.1127 C > T is a pathogenic nonsynonymous mutation located in the tumor necrosis factor (TNF) homology subdomain of exon 8 of the *EDA* gene. c.1133 C > T is a previously reported pathogenic mutation located in different transcripts that is identical to c.1127 C > T (**Figures 3A**–**E**). c.648___683delACCTGGTCCTCCAGGTCCTCCTGGTCCTCAAGGACC (p.216_228delPPGPPGPPGPQGP; NM_001005609) is a nonframeshift deletion mutation located in the collagen subdomain of exon 4 of the *EDA* gene, which was found in family 2 (**Figures 3F–H**).

**Table 2:** *EDA* gene mutations detected in this study.

Number	Patient	Familial/sporadic	Gene	Exon	Mutation type	Nucleotide mutation	Protein alteration	Origin
**1**	Family 1 (III:1.3.6.7.8.9)	Familial	EDA	8	Missense	c.C1127T	p. T376M	Chinese
**2**	Family 1 (I:2;II:2.4.6.8;III:4.10;IV:1)	Familial	EDA	8	Missense	c.C1133T	p. T378M	Chinese
**3**	Family 2 (III:3)	Familial	EDA	4	Nonframeshift deletion	c.648_683del	p.216_228del	Chinese

### ANNOVAR Software Annotation

ANNOVAR annotation indicated that the Polyphen2_HVAR value of c.1127 c > T was 1.0, D. It shows that this mutation is highly damaging to protein structure and function, which is a “probably damaging” mutation. The SIFT value of c.1127 c > T was 0, indicating that this mutation can lead to changes in protein structure or function, which may be a “pathogenic” mutation. c.648_683delACCTGGTCCTCCAGGTCCTCCTGGTCCTCAAGGACC did not obtain the value by annotation.

### Literature Review and Statistical Analysis

We reviewed published papers from PubMed and summarized the novel mutations related to HED. There were 68 novel identified mutations (**Table 3**), among which 57 (83.8%) were *EDA* mutations, excluding unknown genetic forms, mainly with X-linked recessive linkage family inheritance. Eight (11.8%) were *EDAR* mutations, 2 (2.9%) were *EDARADD* mutations, and 1 (1.5%) was a *WNT10A* mutation. Of the 68 mutation, 31 (45.6%) were missense, 23 (33.8%) were deletions, 1 (1.5%) was an indel, and 13 (19.1%) were other types. Genotype–phenotype analysis showed that compared with *EDA* deletion mutations, patients with *EDA* missense mutations had a higher frequency of hypohidrosis (P = 0.021, **Table 4**). There were no other differences in clinical manifestations between the *EDA* mutations and the *EDAR* mutations (**Table 5**).

**Table 3:** Summary of novel gene mutations associated with HED (January 1, 2015–February 3, 2019).

Number	Familial/sporadic	Gene	Exon	Mutation type	Nucleotide mutation	Protein alteration	Origin	Inheritance patterns
1	Familial	*EDA*	—	Deletion	c.954delC	—	Chinese	XLR(Lei et al., 2018)
2	Familial	*EDARADD*	2?	—	c.120 +1G > A (IVS2 +1G > A)	—	South Indian	AR(Chaudhary et al., 2016)
3	Familial	*EDA*	3	INDEL mutation	c.456_468del113insT	p. Arg152_156insdel	Italian	XLR(Callea et al., 2016)
4	Familial	*EDA*	5	Missense	c.659C *>* T	p. P220L	Chinese	XLR(Li et al., 2015)
5	Familial	*EDARADD*	—	Missense	c.367G > A	p. Asp123Asn	German	AD(Wohlfart et al., 2016b)
6	Familial	*EDAR*	—	Splice site mutation	c.730-2 A > G (IVS 8-2 A > G)	—	Iranian	AR(Torkamandi et al., 2016)
7	Familial	*EDA*	intron 3	Splicing mutation	(c.526+1G > A)	—	Chinese	XLR(Liu et al., 2018a)
8	Familial	*EDA*	1	Missense	c.146T > A	p. L49H	Japanese	XLR(Yasuda et al., 2015)
9	Familial	*EDA*	intron 4	—	c.707-1G > A	—	Mexican	XLR(Pina-Aguilar et al., 2018)
10	Familial	*EDA*	4	Frameshift deletion	c.663_697del	p. T221fsX6	Chinese	XLR(He et al., 2018)
11	Familial	*EDA*	4	Frameshift deletion	c.587_615del	p. P196fs X33	Chinese	XLR(He et al., 2018)
12	Familial	*EDA*	7	Missense	c.878 T > G	p. Leu293Arg	Chinese	XLR(He et al., 2018)
13	Sporadic	*EDA*	4	Nonframeshift deletion	c.663_680delTCCTCCTGGTCCTCAAGG	p.222_227delPPGPQG	Egyptian	XLR(Gaczkowska et al., 2016)
14	Familial	*EDA*	—	Missense	c.662G > A	p. Gly221Asp	Chinese	XLR(Zeng et al., 2016)
15	Familial	*WNT10A*	—	Missense	c.354T > G	p. Tyr118*	Chinese	AR(Zeng et al., 2016)
16	Familial	*EDAR*	12	Frameshift mutation	c.1193_1194delTT	p. Phe398X	Italian	AD(Callea et al., 2015)
17	Familial	*EDA*	8	Missense	c.878T > G	p. Leu293Arg	Chinese	XLR(Xue et al., 2015)
18	Familial	*EDA*	1	Frameshift mutation	c.172-173insGG	—	Chinese	XLR(Lin et al., 2017)
19	Familial	*EDA*	—	Missense	c.1073A > T	Q358 L	Chinese	XLR(Liu et al., 2018b)
20	Familial	*EDA*	8;9	Deletion	c.682_683delCCinA	P228Tfs*52	Chinese	XLR(Liu et al., 2018b)
21	Unknown	*EDA*	—	Duplication	c.64_71dup	p. Cys25AlafsX35	Unknown	Unknown(Wohlfart et al., 2016a)
22	Unknown	*EDA*	2	Duplication	c.397-5858_502+3441dup	p. Gly168AspfsX10	Unknown	Unknown(Wohlfart et al., 2016a)
23	Unknown	*EDA*	—	Deletion	c.467_468del	p. Arg156GlnfsX2	Unknown	Unknown(Wohlfart et al., 2016a)
24	Unknown	*EDA*	—	Missense	c.601G > T	p. Gly201X	Unknown	Unknown(Wohlfart et al., 2016a)
25	Unknown	*EDA*	—	Missense	c.608C > T	p. Pro203Leu	Unknown	Unknown(Wohlfart et al., 2016a)
26	Unknown	*EDA*	—	Splice site modification	c.793G > T	p. Asp265Tyr	Unknown	Unknown(Wohlfart et al., 2016a)
27	Unknown	*EDA*	—	Missense	c.935T > A	p. Ile312Asn	Unknown	Unknown(Wohlfart et al., 2016a)
28	Unknown	*EDA*	—	Deletion	c.252del	p. Gly85AlafsX6	Unknown	Unknown(Wohlfart et al., 2016a)
29	Unknown	*EDA*	—	Deletion	c.376_379del	p. Asp126ProfsX10	Unknown	Unknown(Wohlfart et al., 2016a)
30	Unknown	*EDA*	—	Splice site modification	c.396+5G > A	—	Unknown	Unknown(Wohlfart et al., 2016a)
31	Unknown	*EDA*	2	Duplication	c.397-6070_502+3112dup	p. Gly168AspfsX10	Unknown	Unknown(Wohlfart et al., 2016a)
32	Unknown	*EDA*	2	Deletion	c.397-? _502+? del	p. Met133AlafsX112	Unknown	Unknown(Wohlfart et al., 2016a)
33	Unknown	*EDA*	4–6	Deletion	c.527-3066_793+1017del-ins8	p. Lys177ValfsX17	Unknown	Unknown(Wohlfart et al., 2016a)
34	Unknown	*EDA*	—	Deletion	c.542_577del	p. Gly180_Pro191del	Unknown	Unknown(Wohlfart et al., 2016a)
35	Unknown	*EDA*	—	Splice site modification	c.707-13T4G	—	Unknown	Unknown(Wohlfart et al., 2016a)
36	Unknown	*EDA*	—	Missense	c.1009G > T	p. Glu337X	Unknown	Unknown(Wohlfart et al., 2016a)
37	Unknown	*EDA*	—	Missense	c.1075A > T	p. Lys359X	Unknown	Unknown(Wohlfart et al., 2016a)
38	Unknown	*EDA*	—	Missense	c.1112T > A	p. Ile371Asn	Unknown	Unknown(Wohlfart et al., 2016a)
39	Unknown	*EDA1R*	—	Deletion	c.126del	p. Leu43CysfsX60	Unknown	Unknown(Wohlfart et al., 2016a)
40	Unknown	*EDA1R*	—	Deletion	c.486del	p. Ser163ArgfsX26	Unknown	Unknown(Wohlfart et al., 2016a)
41	Unknown	*EDA1R*	—	Deletion	c.1146_1149del	p. Leu383ArgfsX8	Unknown	Unknown(Wohlfart et al., 2016a)
42	Unknown	*EDA1R*	—	Deletion	c.1169del	p. Gly390AlafsX2	Unknown	Unknown(Wohlfart et al., 2016a)
43	Familial	*EDA*	1	—	c.84_85insC	p. G29fs*69	Mexican	XLR(Monroy-Jaramillo et al., 2017)
44	Familial	*EDA*	1	Missense	c.1116C > G	p. N372K	Mexican	XLR(Monroy-Jaramillo et al., 2017)
45	Familial	*EDA*	1	Nonsense	c.106G > T	p. E36Ter	Mexican	AR(Salas-Alanis et al., 2015)
46	Familial	*EDA*	3	Missense	c.448G > A	p. E150K	Mexican	AR(Salas-Alanis et al., 2015)
47	Familial	*EDA*	4	Deletion	c.Del 546-581	p.183-194del	Mexican	AR(Salas-Alanis et al., 2015)
48	Familial	*EDA*	5	Missense	c.574G > C	p. G192R	Mexican	AR(Salas-Alanis et al., 2015)
49	Familial	*EDA*	6	Splice site	c.793+1 G > C	—	Mexican	AR(Salas-Alanis et al., 2015)
50	Familial	*EDA*	7	Deletion	Del 887-900	p.297-301delFSx4	Mexican	AR(Salas-Alanis et al., 2015)
51	Familial	*EDA*	8	Missense	c.894G > C	p. G299R	Mexican	AR(Salas-Alanis et al., 2015)
52	Familial	*EDA*	9	Missense	c.1037G > A	p.C346Y	Mexican	AR(Salas-Alanis et al., 2015)
53	Familial	*EDA*	9	Missense	c.1038C > G	p.C346W	Mexican	AR(Salas-Alanis et al., 2015)
54	Familial	*EDA*	9	Missense	c.1049G > A	p. G350D	Mexican	AR(Salas-Alanis et al., 2015)
55	Familial	*EDA*	6	Deletion	c.742_793del	p.P248_D265del I248fsX261	Chinese	XLR(Xu et al., 2017)
56	Familial	*EDA*	—	Missense	c.852T > G	p. Phe284Leu	Chinese	XLR(Zeng et al., 2015)
57	Familial	*EDA*	—	Missense	c.1051G > T	p. Val351Phe	Chinese	XLR(Zeng et al., 2017)
58	Familial	*EDA1*	1	Missense	c.409T > C	p. Leu56-Pro	Mexican	XLR(Pozo-Molina et al., 2015)
59	Familial	*EDAR*	—	Missense	c.1249C > T	p. Gln417*	Pakistani	AR(Ahmad et al., 2018)
60	Familial	*EDAR*	12	Missense	c.1190T > A	p. L397H	Indian	AD(Chaudhary et al., 2017)
61	Unknown	*EDA*	7	Deletion	c.915_922del	p. Ser305Argfs*9	Japanese	XLR (Nakajima et al., 2019)
62	Familial	*EDA*	4	Deletion	c.639delT	p. Met214Trpfs*66	Japanese	XLR (Nakajima et al., 2019)
63	Familial	*EDA*	—	Splice site	c.925-2A > G	—	Chinese	?(Feng et al., 2018)
64	Familial	*EDA*	3	Nonsense	c.511A > T	p. Lys171*	Japanese	?(Okita et al., 2019)
65	Familial	*EDA*	1	Deletion	c.5delG	p. Gly2Alafs*55	Japanese	?(Okita et al., 2019)
66	Familial	*EDA*	1	Missense	c.158T > A	p. L53H	Italian	XLR(Savasta et al., 2017)
67	Sporadic	*EDA*	—	Missense	c.917A > G	p. Q306R	Japanese	XLR(Miyake et al., 2017)
68	Familial	*EDA*	—	Deletion	c.302_303delCC	p. Pro101HisfsX11	Chinese	XLR(Ma et al., 2018)

**Table 4:** Comparison of features in novel *EDA* gene missense and deletion patients.

Features	Missense (n = 34)	Deletion (n = 12)	Method	*P*
Facial features	27/7	10/2	**χ^2^** test	1
Hypotrichosis	31/3	10/2	**χ^2^** test	0.833
Hypohidrosis	34/0	11/1	Fisher's test	0.021
Hypodontia or Oligodontia	29/5	9/3	**χ^2^** test	1

The number after “/” indicates the patients without the feature. The “n” indicates the total number of patients.

**Table 5:** Comparison of features in Novel *EDA* gene mutations (missense and deletion) and *EDAR* gene mutations patients.

Features	*EDA* (n = 46)	*EDAR* (n = 10)	Method	*P*
Facial features	37/9	10/0	Fisher's test	0.668
hypotrichosis	41/5	10/0	Fisher's test	0.333
Hypohidrosis	45/1	10/0	Fisher's test	0.578
Hypodontia or oligodontia	38/8	10/0	Fisher's test	1.000

The number after “/” indicates the patients without the feature. The “n” indicates the total number of patients.

## Discussion

The *EDA* gene contains 12 exons, with at least nine different transcripts produced by alternative splicing (Liu et al., 2018a). It is a trimeric type II transmembrane protein located at Xq12-q13.1 that contains an intracellular domain, a transmembrane domain, a furin subdomain, a 19-repeat Gly-X-Y collagenous domain, a TNF homology subdomain, and a cysteine-rich C-terminal domain (Schneider et al., 2001). Approximately half of HED patients have *EDA* gene mutations (Cluzeau et al., 2011) that may impact protein function, which subsequently affects the ectodysplasin/nuclear factor-κB signaling pathway. In addition to *EDA*, the literature also associated HED with mutations in the *EDAR*, *EDARADD*, *TRAF6*, *WNT10A*, and *NEMO* genes (Cluzeau et al., 2011). Most of the mutations in these genes caused HED by affecting the ectodysplasin/NF-κB or Wnt/β-catenin pathways related to the normal development of ectodermal structures (Clauss et al., 2008; Cluzeau et al., 2011).

A review (Huang et al., 2015) of the NCBI ClinVar database and published articles stated that at least 82 pathogenic mutations in *EDA* genes were associated with HED. Among these, 41 (50%) were missense), 13 (15.9%) were deletions, 12 (14.6%) were nonsense, and 9 (11%) were frameshift. Only one (1%) intronic mutation was reported. In addition, 31 (37.8%) mutations in the TNF homology subdomain domain 18 (22%) mutations in the collagen subdomain domain, 6 (7.3%) in the transmembrane domain, and 6 (7.3%) in the furin subdomain. This suggests that *EDA* mutations play a critical role in HED.

Here we described two Chinese Han HED families with two *EDA* mutations. In family 1, whole-exome and Sanger sequencing revealed a mutation (c.1127 C > T), in the TNF homology subdomain that converts a cytosine to thymine, and the corresponding amino acid is changed from threonine to methionine (p.T376M). We also found a reported pathogenic mutation (c.1133 C > T) (Wang et al., 2014; Ngoc et al., 2018) in family 1 by Sanger sequencing, but it was the same mutation as c.1127 C > T in different transcripts. Since the TNF homology subdomain plays a key role in the ligand homotrimerization and receptor binding, the *EDA* gene may be unable to bind to its ligand (*EDAR*) in members of this family. The c.1127 C > T (or c.1133 C > T) hemizygous male patients in this family are more severely affected, while heterozygous females show only mild to moderate degrees of typical HED features. The proband of this family had a healthy father, mother, and brother. Pedigree analysis showed that six males in four successive generations were affected, and eight females in four successive generations showed normal or moderate features, indicating an X-linked recessive pattern in this family. In addition, ANNOVAR annotation indicated that c.1127 C > T is a “probably damaging” mutation. Therefore, a successful genetic diagnosis was made in family 1.

In family 2, one etiological mutation was found in the *EDA* gene coding region. An in-frame deletion was located in the short collagenous domain c.648_683delACCTGGTCCTCCAGGTCCTCCTGGTCCTCAAGGACC (p.216_228delPPGPPGPPGPQGP). This 36-bp c.648_683delACCTGGTCCTCCAGGTCCTCCTGGTCCTCAAGGACC deletion mutation removes 13 amino acids (p.216_228delPPGPPGPPGPQGP). Gene sequencing revealed that the mutation was only in the proband. Clinical examination revealed that he had dry skin, decreased sweating, sparse hair, missing teeth, frontal bossing, prominent lips, and periorbital wrinkling; patchy pigmentation and depigmentation could be seen in his trunk and limbs. His parents were normal, and c.648_683delACCTGGTCCTCCAGGTCCTCCTGGTCCTCAAGGACC did not have a high value based on ANNOVAR annotation. However, we hypothesize that this deletion can shorten the collagen domain in the encoded protein, which may disrupt the domain's functions. Pedigree analysis indicated that the proband may have an X-linked recessive pattern, but further follow-up is needed.

In the published work review, 68 novel identified mutations were summarized (**Table 3**). Over 80% of mutations were found in *EDA* (mainly missense), and more than 40% of 68 mutations were missense. Although patients with HED always have similar clinical features, deviations in the degree of severity are observed (Schneider et al., 2011; Zhang et al., 2011; Burger et al., 2014; Wohlfart et al., 2016a). Zeng et al. (2015) and Gaczkowska et al. (2016) found that HED patients with truncating *EDA* mutations tend to lose more permanent teeth than patients with non-truncating mutations, while missense mutation patients likely lose fewer permanent teeth than patients with other types of mutations. Our study revealed that patients with *EDA* missense mutations had a higher frequency of hypohidrosis (P = 0.021). However, the results in our study may be attributable to the small sample size. Further studies with larger numbers of patients are required to clarify whether there is a clear association between specific mutations and different manifestations.

In summary, we identified two *EDA* gene mutations in two Chinese Han families with X-linked HED and provided genetic counseling. We hope that our findings will be helpful for genetic counseling, carrier detection, prenatal diagnosis, and clinical practice. However, further studies on genotype–phenotype correlations in HED patients are still needed.

## Data Availability Statement

The datasets generated analayzed for this study can be found in the SRA accession: PRJNA596941.

## Ethics Statement

The studies involving human participants were reviewed and approved by the Ethical Review Committee of Anhui Medical University. The patients/participants provided their written informed consent to participate in this study. Written informed consent was obtained from the individual(s), and minor(s)' legal guardian/next of kin, for the publication of any potentially identifiable images or data included in this article.

## Author Contributions

All authors listed have made a substantial, direct, and intellectual contribution to the work, and approved it for publication.

## Conflict of Interest

The authors declare that the research was conducted in the absence of any commercial or financial relationships that could be construed as a potential conflict of interest.
